# Photocurable Polymers for Ion Selective Field Effect Transistors. 20 Years of Applications

**DOI:** 10.3390/s90907097

**Published:** 2009-09-07

**Authors:** Natalia Abramova, Andrei Bratov

**Affiliations:** Instituto de Microelectrónica de Barcelona (IMB-CNM-CSIC), Campus UAB, 08193 Bellaterra, Barcelona, Spain; E-Mail: andrei.bratov@imb-cnm.csic.es

**Keywords:** ISFET, photocurable, polymers, membrane, chemical sensor

## Abstract

Application of photocurable polymers for encapsulation of ion selective field effect transistors (ISFET) and for membrane formation in chemical sensitive field effect transistors (ChemFET) during the last 20 years is discussed. From a technological point of view these materials are quite interesting because they allow the use of standard photo-lithographic processes, which reduces significantly the time required for sensor encapsulation and membrane deposition and the amount of manual work required for this, all items of importance for sensor mass production. Problems associated with the application of this kind of polymers in sensors are analysed and estimation of future trends in this field of research are presented.

## 1. Introduction

The field of chemical sensors based on microelectronic devices has been the topic of considerable research in the last decades and has been largely focused on ion selective field effect transistors. This interest arises from certain advantages of ISFETs over conventional ion-selective electrodes (ISE) such as a small size and solid nature, short response times and low output impedance and the possibility of mass fabrication. Other features such as the integration of compensation and data processing circuits in the same chip also offered new perspectives for these sensors.

The response mechanisms of ISFET-based sensors are based on the electrochemical phenomena occurring within the chemically sensitive membrane placed on top of the transistor gate [1] and on electrical transduction of the signal by this semiconductor device. To exploit the advantages offered by modern technologies in a fabrication of solid-state chemical sensors based on ISFETs new requirements are imposed on any potential matrix for ion sensor membranes, namely the adhesion of membranes to a solid substrate and the compatibility of any ion-sensitive membrane deposition process with microelectronic technology.

Finally, the main obstacle preventing widespread production of ISFETs and chemically sensitive field effect transistors (ChemFETs) is the lack of a good sensor chip encapsulation procedure. Though ISFETs are now commercially available, most encapsulation procedures used are done manually.

To resolve these problems the most feasible approach from technological point of view is to use polymer materials that may be cured by UV light. The use of photocured polymeric systems permits one to employ a standard photolithographic process, which reduces significantly the time of membrane deposition and facilitates the encapsulation process reducing the manual work required for it, items that are all important for sensor mass production.

## 2. Encapsulation of ISFET Sensors

Packaging of chemical microsensors based on ISFET devices produced by silicon planar technology is one of the most important and critical steps in their whole realisation process. For industrial applications a complete automatization of the fabrication process is necessary. Several techniques have been developed for this by applying microtechnologies such as photolithography [2], electrochemical deposition [3], anodic bonding [4] and micromachining [5].

The most promising from a technological point of view is the application of photosensitive encapsulants. Among the materials used are polyimide covered by a standard photoresist [6] and photocurable epoxy acrylate [7]. Polyimide is widely used in semiconductor and packaging technologies because of its high thermal stability and good insulation properties. The problem in applying photosensitive polyimides for encapsulation is that it is difficult to deposit and to selectively etch thick (>20 μm) films of this material [8]. Moreover it is not chemically stable in highly basic solutions.

Multifunctional acrylates are widely used in different industries for production of UV cured coatings. These materials are usually characterised by their high reactivity and give insulating coatings with good weatherability and high resistance to solvents. Acrylates upon exposure to UV undergo polymerisation via radical or cationic mechanisms, depending on a photoinitiator used. Radical polymerisation, being a faster process, is preferred in most cases. The polymer layer after being applied to a wire bonded sensor glued to some substrate can be patterned using traditional photolithography techniques. The properties of the resulting encapsulant will depend on the chemical structure of the prepolymer used. In choosing a suitable prepolymer it must be taken into consideration that is has to satisfy certain requirements, namely:

- Good chemical resistance in highly acidic or alkaline media
- Low permeability for any kinds of chemical compounds
- Good adhesion to a solid substrate (silicon, silicon oxide or silicon nitride)
- Easy processing
- High electrical resistance for preventing leakage current
- Mechanical strength and low shrinkage
- Biocompatibility in the case of biomedical applications

**Figure 1.**
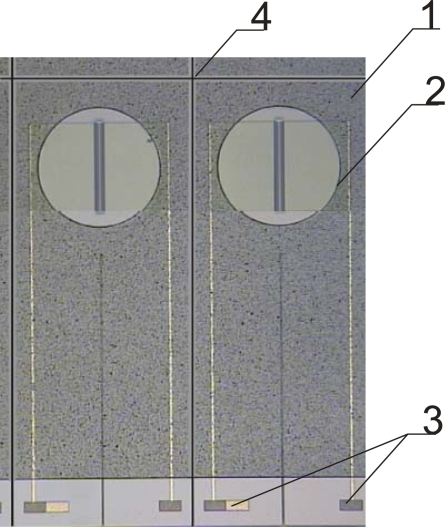
ISFETs wafer with the thin layer of photocurable encapsulant – 1, opening over the gate region – 2, contact pads – 3 and scribing lines −4.

Investigations [9,10] have shown that acrylate derivatives of aliphatic urethane and bisphenol A (epoxy) resins are promising materials for ISFET sensor encapsulation, which along with good insulating properties posses good chemical resistance. A multilayer deposition method was developed where to enhance the adhesion of a thick polymer film covering the mounted and wire bonded chip a first thin layer of photocurable encapsulant is initially deposited directly on a wafer (Figure 1).

The layer (1) is patterned to open precisely the gate (2), contact pads (3) and scribing lines (4).

**Figure 2.**
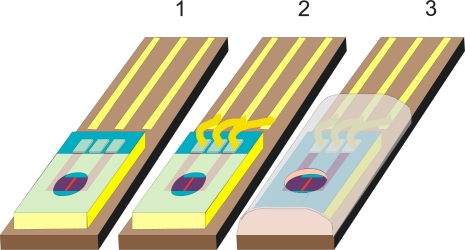
ISFET sensor mounting and encapsulation process (explanation in the text).

**Figure 3.**
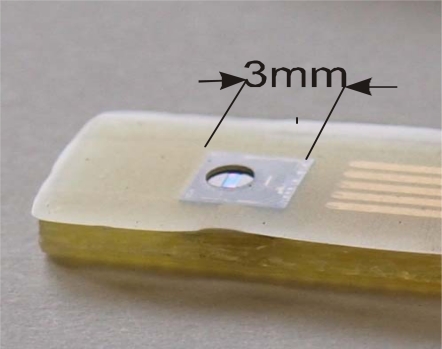
Photograph of a wire bonded and encapsulated ISFET chip.

An ISFET sensor mounting and encapsulation process is presented schematically on Figure 2. After cutting the wafer the chips are glued to the PCB plate with contact lines (1) and are wire bonded (2) to provide a contact to source, drain and silicon substrate. The plates with wire bonded sensors are fixed in a mould where they are covered with a thick (100–200 μm) layer of the encapsulant. After exposure to UV through a mask that protects the gate region, the polymer is developed leaving the opening in the encapsulant (3). Figure 3 presents the photograph of the ISFET chip encapsulated by this method. These types of sensors are provided by D + T Microelectrónica (Spain) for special applications according to user requirements.

## 3. Membrane Formation

The best known method of ISFET membrane formation comes from traditional ion selective electrodes and is based on using a polymer matrix which is deposited over an ISFET gate and contains the required ion active components, like ionophore, plasticizer and lipophilic additives. Achievements in development of traditional ISE with liquid inner contact resulted in hundreds of different membrane compositions that can be used as well in case of ISFETs

The most common polymer matrix for membrane ISEs is polyvinyl chloride (PVC). Unfortunately, its use in ISFET-based sensors is limited due to poor adhesion of PVC to a solid support which leads to peeling of membranes, producing drift in sensor parameters and short sensor operational lifetimes. Another drawback is the manual deposition of membranes by solvent casting which requires long curing times under ambient conditions with controlled partial pressure of the solvent. And the last problem that face PVC-based membrane ISFETs is the leaching of membrane components from rather thin (30–100 μm) membranes, resulting in changes in the membrane composition and deterioration of sensor parameters.

Photocurable polymers as an alternative material for membrane matrices for ISFETs, solid contact electrode (SCE) and coated wire electrode (CWE) were introduced by different research groups in the middle of 1980s, and early 1990s. Bibliographic data on application of photocurable polymers for chemical and ion sensor development are summarized in Table 1. Among the different photocurable polymeric systems acrylates and methacrylates are the most commonly used. Coated wire electrodes selective to calcium and potassium ions with membranes based on commercial epoxyacrylates were studied by an Australian group [11,13–15]. Later the same polymer was used for the development of a Ca-selective electrode [12] and for a nitrate sensitive ISFET [34,35], but with different polymerisation schemes. This polymer was also applied to fabricate a six sensor array of coated-wire electrodes for use in a portable flow-injection analyzer [17].

In 1994 Bratov *et al*. [57] demonstrated that polyurethane based photocurable polymers may be regarded as an appropriate matrix for ISFETs ion selective membranes. This polymer matrix contains a small amount of anionic impurities that cause intrinsic cationic permselectivity [19]. Being compatible with various plasticizers traditionally used in ion selective membrane formulations, it may be used to make different sensors selective to cations [19–21] as well as anions [22–24]. For proper functioning the membrane with cation selective neutral carrier as an ionophore should contain at least 35% of a suitable plasticizer and small amounts of a salt with a highly lipophilic anion (K-TpClPB). Polyurethane polymers showed excellent adhesion to silylated silicon oxide surface giving membranes with a long (more than 6 months) life-time during constant contact with solutions.

Polysiloxane was used not only as a supporting material for ISFETs but as a polymer that allowed research groups from the University of Neuchatel [36–39] and the University of Twente [38] to covalently attach an ionophore and anionic sites to the membrane matrix. Among other photocurable materials styrene-vinylbenzol copolymer [51] and different types of poly(vinyl alcohol) (PVA) [52–56] were reported. PVA as well as polyacrylamide [30], due to their hydrophilic nature, were successfully applied for enzymatic biosensors.

The use of photocured polymeric systems permits the use of standard photolithographic processes to form ion-sensitive membranes on all the devices on a wafer level. This may be beneficial for mass production of ion sensors. However, membranes cover only a small portion of the wafer area and most of the deposited pre-polymer composition will be washed away during the development stage, which may be regarded as a certain disadvantage taking into consideration the high cost of ionophores. A more economical solution to this problem is the use of microdispensers with which one can apply a certain volume of a pre-polymer mixture to the well formed by encapsulating layer over the ISFET gate region. This technique may be used to deposit membranes directly on a wafer or on individual encapsulated sensors, as presented in Figure 3. In this case it is easy to form thick membranes of 200–300 μm which may have a longer lifetime. By above method ion-sensitive membranes of different types may were formed on an integrated multi-sensor chip [58].

**Figure 4.**
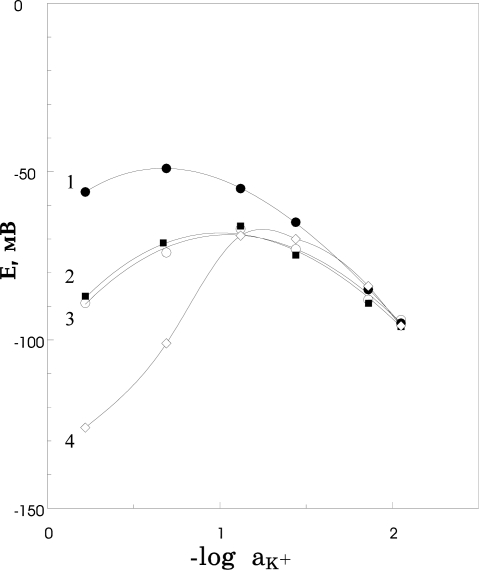
Calibration curves for polyurethane membranes containing K-TpClPB and a plasticizer in solutions of highly lipophilic anion (KCNS). The membranes differ in the UV exposure time used for polymerisation: 1–2 minutes of irradiation; 2–10 minutes; 3–20 minutes; 4-membrane without K-TpClPB and with 2 minutes of irradiation.

One of the problems that may arise in the case of photocurable polymer membranes is a photobleaching of the membrane components upon extended UV exposure during the polymerisation and encapsulation processes. It is known that the stability of tetraphenylborate ion is limited, especially in the presence of acids and oxidizing agents and under UV illumination [59]. Decomposition of tetraphenylborate derivatives takes place with the consumption of a proton, giving as a result neutral products. This means that the concentration of tetrakis(*p*-chlorophenyl)borate ion that is normally used for an ion selective membrane in order to reduce the resistance of the membrane and to obtain the better selectivity may depend on the time of UV exposure. The extent of interference of highly lipophilic anions (CNS- for example) in solution may be used as an indicator for the K-TpClPB content in the membrane, as shown in Figure 4, where the calibration curves of sensors with membranes containing plasticizer and K-TpClPB and polymerised with different exposure times are presented. For comparison the calibration curve for membrane without lipophilic anion is given too [60]. From Figure 4 it is clear that with increase of the exposure time the cationic response of the membrane switches to anionic at much lower concentrations of KCNS and selectivity of the sensor decreases.

Another problem that photocurable polymer membrane matrices may suffer is the impossibility to use some plasticizers or ionophores, especially in the case of radical initiation of polymerisation because they can inhibit this type of reaction. No polymerisation occurred when 2-nitrophenyl octyl ether (NPOE) or 4-nitrophenyl phenyl ether (NPPE), two of the most common plasticizers for bivalent ions like Ca or Mg, are used in membrane compositions [13,19]. The presence of porphyrin, which acts as an ionophore in many anion selective membrane compositions, in case of urethane diacrylate also completely inhibits the photopolymerisation process [22]. This problem may be partly resolved by changing the type of initiation of photopolymerisation from radical to cationic [12,34] that is less susceptible to the inhibition by these substances. As a photoinitiator of cationic polymerisation process a mixture of quinone derivatives and phenyliodonium salt is used. It must be taken into consideration that introduction of photoinitiators may affect analytical parameters of sensors. It is well known that a photoinitiator is not totally consumed in the reaction [61] and though its concentration in the membranes is not very high, it is comparable with the concentration of an ionophore and lipophilic additives (1–2 %w/w). This may result in that the products of fragmentation and unreacted substances alter the ion-selective properties of the resulting membranes. Usually the selectivity of membranes that were cured via cationic way is not very high [12,17,34].

Attempts to prevent the leaching of ion active components from thin (30–100 μm) membranes began with the synthesis of a so called copolymerisable plasticizer [62]. More lately “plasticizer-free” [36,37] or “self-plasticizing” [38,39] ion-selective membranes have been introduced. This work was performed by the group from University of Cambridge (UK) [43,44,63,64] who studied different copolymers based on acrylate and methacrylate systems. Another plasticizer-free polymer membrane composition based on methyl methacrylate and decyl methacrylate (MMA-DMA) copolymer was proposed by the Bekker group [65,66]. The last two works describe a thermally initiated free radical polymerisation method but the opportunity of photocuring of such systems may be mentioned. The use of commercial acrylates (Epocryl DRH 370, ACE and Cardura from Shell, Amsterdam) by the group at the University of Twente was not successful and the resulting K-selective ISFETs demonstrated low sensitivity [67]. More interesting results were obtained by incorporation of methacrylate moieties in the polysiloxane by Reinhoudt *et al*. [37]. However, the synthesis of these siloxane polymers seems to require much effort. To avoid this complicated synthesis the use of a commercial oligosiloxane and a polar methacrylate was suggested [46]. In this case K- and Ca-sensitive ISFETs with stable electro-analytical parameters were reported.

It must be noted that the absence of a free plasticizer within the membrane matrix may cause two problems. Firstly, the solubility of an ionophore and lipophilic additives in the membrane mixture will be decreased, which may seriously affect the sensor parameters like sensitivity, selectivity and limit of detection. Secondly, this will affect the mobility of charged species within the membrane phase which will result in a very high membrane impedance (50–200 MΩ). In this case it is impossible to use a traditional ISFET-meter circuitry where changes of polarisation potential are registered under constant drain current conditions. The problem arises from the fact that membrane resistance in series with the capacitor of an ISFET gate have a large time constant which causes a delay in the drain current response when the gate potential is changed. In traditional ISFET-meter circuitry the drain current is maintained at a constant value by means of an operation amplifier, which directly controls the applied gate bias potential with negative feedback loop. However, at high values of membrane resistance, more than 10 MΩ, and with a delay in drain current response the circuit starts to oscillate [22]. Introduction of lipophilic additives, like K-TpClPhB and tetradodecylammonium tetrakis(4-chlorophenyl)borate (ETH 500) does not always help to reduce significantly the resistance [23].

Finally, another problem must be addressed. Due to the fact that in many cases commercial oligomers and not individual substances of known purity grade are used to prepare polymer membrane compositions it is difficult to expect good repeatability of the results when different batches of oligomers are used. These normally contain some additives of organic and inorganic nature originating from the industrial production or laboratory synthesis processes. Usually it is impossible to predict how these impurities will affect the analytical properties of ion selective membranes and to guarantee that their behaviour will be repeatable. This fact may explain why sometimes controversial results are published. Especially it concerns the selectivity of ion-selective membranes, a parameter that is very sensitive to the presence of ionic sites. In case of photocurable polymers this influence may be more pronounced because in addition to the impurities of polymers and cross-linkers the products of photobleaching may affect the sensor behaviour.

## 4. Conclusions and Perspectives

As a general conclusion, it is possible to note that nowadays scientific interest in ISFET sensors appears to be in decline. This is true not only for ChemFETs with photocurable membranes, but for all types of ISFETs. This can be explained by the fact that these devices are already quite well studied, suitable for various applications and with the fabrication technology established, they face more marketing problems than scientific ones. On the other hand, many groups which have been involved in the research on ISFETs throughout the years have changed research topics because financial support is probably more easily attracted for other, newer areas of research [68]. In any case, it is expected that in the future the on-line monitoring of industrial processes will become an important market for ISFET applications. In recent decades, the interest in quality control of food, water, products for human use and anything that could have adverse effects on the environment or human health has increased considerably. To date one of the best approaches to the quality control is provided by use of chemical sensor arrays with posterior multivariate analysis of their response. One of the systems that exists on the market now consists of an array of seven cross-selective sensors based on ISFETs for measuring organic as well as inorganic compounds, an autosampler, a software package and an electronic unit. This system under the name of α-ASTREE is produced by the French company Alpha M.O.S. [69] and has been evaluated in food applications such as characterisation of flavours, quantitative analysis and quality control. According to the company’s publicity the sensors can be fabricated with different membranes specially focused to the particular application demands of end-users.

One of the most important drawbacks of these arrays (or the so called “electronic tongues” in case of the analysis of liquids) reported until now is that normally they are composed by the same type of the sensors, either potentiometric, voltammetric or interdigitated electrodes [70]. This implies that a limited amount of data that can obtained with this analysis method. The application of microelectronic technologies permits monolithic integration of different electrochemical sensors using silicon technology. In this case it is possible to develop silicon chips with multi-sensor arrays that may include ion selective field effect transistors with various type of membranes and interdigitated electrodes used to measure the conductivity, redox potential or impedance parameters, amperometric sensors and other type of the sensors like temperature diode, for example [71]. As it was shown, this type of the sensors array can be successfully applied for mineral water analysis [72–73], as well as for grape juice and wine analysis [74]. It permits not only to distinguish the wines according to the grape variety and the vintage year, but also can be used for quantitative prediction of several sample parameters [74].

Another type of semiconductor based chemical sensors where application of photocurable membranes may be promising is light-addressable potentiometric sensors (LAPS), where the same field effect principle as in case of ISFET is used [47]. The advantage of the LAPS technique is that an arbitrary position on the sensing surface of the LAPS can be independently accessed with a light probe, e.g., scanning laser beam. This “light-addressability” facilitates its application to integrated multi-LAPS, in which different parts of the sensing surface are modified with different type of membranes (ion-selective, enzymatic, etc) [75]. These different microelectronic sensors combined together with microactuators (valve, pumps, channels, etc) and micro flow cells form analytical systems of next generation like μTAS (micro total analysis system) or lab-on-a-chip devices.

## Figures and Tables

**Table 1.** Photocurable applied polymers as membrane matrices for chemical sensors.

**Type of polymer**	**Analyte**	**Type of sensor**	**Reference**
**ACRYLATES**			
Bisphenol A epoxyacrylate (Ebecryl 150)	Ca^2+^	SCE	[11]

Bisphenol A epoxyacrylate (Ebecryl 600)	Ca^2+^	SCE, CWE	[12–13]

	K^+^	SCE	[14–15]

	Li^+^	SCE	[16]

	NH_4_^+^, Ca^2+^, pH, K^+^, Cl^−^, NO_3_^−^	CWE	[17]

Epocryl DRH 370	K^+^	ISFET	[18]

Aliphatic urethane diacrylate (Ebecryl 270)	K^+^, NH_4_^+^, Ca^2+^	ISFET	[19],[20],[21]

	Cl^−^, Na^+^	ISFET	[22],[23]

	CO_3_^2−^	ISE	[24]

	pH	SCE	[25]

	monochloroacetate	SCE	[26]

	urea	ISFET	[27]

	glucose	Microelectrode	[28]

	anionic surfactants	ISFET	[29]

Polyacrylamide	Glucose, urea	ISFET	[30]

Dodecyl acrylate	Cl^−^, NO_3_^−^, ClO_4_	Optical sensor	[31]

Acrylic acid and acrylonitrile	Reference electrode	SCE	[32]

Isodecylacrylate and acrylonitrile	Na	SCE	[33]

**METHACRYLATES**			
Butyl methacrylate	Ca^2+^	ISFET	[2]

Bisphenol A-bis(2-hydroxypropyl- methacrylate)	NO_3_^−^	SCE, ISFET	[34–35]

Polysiloxanes	Ca^2+^	ISFET	[36]

	K^+^, NO_3_^−^	ISFET	[37–39]

Butyl-, nonyl-, 1,4-butanduol-, 1,6-hexandiol methacrylates	Ca^2+^	ISFET	[40]

Bisphenol A-diglicidyletherdi-methacrylate	NO_3_^−^, BF_4_, dicyanoureate, dicyanoargentate	SCE	[41]

	K^+^, Ca^2+^	ISFET	[42]

Metyl-, *n*-butyl methacrylates	K^+^, Na^+^, pH, Ca^2+^	SCE	[43,44]

Methyl-, decyl methacrylate	K^+^	Optical sensor	[45]

Oligosiloxane methacrylate	K^+^, Ca^2+^	ISFET, SCE	[46]

Methyl-, butyl-, glycidyl methacrylates	K^+^, Ca^2+^, Cs^+^, Li, Mg^2+^	LAPS	[47]

Hydroxyethyl methacrylate-co-methacrylic acid	pH	Holographic pH sensor	[48]

Polymethacrylate	glucose	SCE	[49]

Glycidyl methacrylate	Urea, acetylcholine, butyrylcholine, Cd^2+^	LAPS	[50]

**OTHERS**			
Styrene-vinylbenzol	K^+^	ISFET	[51]
Poly(vinyl alcohol)	Glucose, urea	ISFET	[52]

PVA-SbQ*	Glucose, sucrose	ISFET	[53]

	Urea, trichlorfon,	ISFET	[54–56]

ISE – conventional ion selective electrode, SCE –solid contact electrodes, CWE – coated wire electrode, LAPS – light addressable potentiometric sensor,

* PVA-SbQ – polyvinyl alcohol functionalised with methyl pyridinium methyl silphate
